# Time Matters: Time Perspectives Predict Intertemporal Prosocial Preferences

**DOI:** 10.3390/bs13070590

**Published:** 2023-07-14

**Authors:** Teng Lu, Dapeng Liang, Mei Hong

**Affiliations:** School of Management, Harbin Institute of Technology, 92 Xidazhi Street, Nangang District, Harbin 150001, China

**Keywords:** intertemporal prosocial discounting, dictator game, time perspective, present impulsive, personality

## Abstract

The study utilizes the Chinese version of the Zimbardo Time Perspective Inventory (ZTPI-C) and a novelty intertemporal prosocial discounting paradigm to explore the preferences of individuals with the Present Impulsive Time Perspective (PITP) and the Future Time Perspective (FTP) in intertemporal prosocial choices, and uncovers the cognitive mechanisms underpinning intertemporal altruism from the personality traits. The findings revealed: (1) The donation behaviors of both groups decreased as time delay rose, aligning with the hyperbolic model. (2) PITP individuals had significantly higher discount rates than those with FTP, and the scores of FTP individuals on the “Future” dimension of the ZTPI-C were positively correlated with the amount of money they were willing to forgo. These results suggest that time perspective, as a stable personality trait, can predict individuals’ intertemporal prosocial preferences. Our research enriches the theory of intertemporal choices and extends the Perceived-time–based model (PTBM) to the domain of intertemporal social preferences.

## 1. Introduction

Time and value are fundamental dimensions of intertemporal decision-making [1]. A commonly observed bias among economic decision-makers is the predilection for immediate satisfaction, which enhances impatience during present-influencing decisions [2]. Evidence supporting this observation comes from various circumstances, such as procrastination [3], health [4], and financial behaviors [5]. These examples uphold the assertion of inconsistency in intertemporal decisions. However, intertemporal trade-offs also feature prominently in social contexts where an individual’s well-being potentially conflicts with others’ [6]. For instance, charitable donations or pro-environmental actions involve immediate personal sacrifices for deferred benefits to others. Repetitive interactions, such as reciprocal exchanges, naturally involve time and space considerations. As a result, the inherent intertemporal nature of prosocial choices requires individuals to weigh their benefits against those of others at different points in time [7].

The concept of “time perspective”, a reflection of individuals’ differing perceptions of time, has been proposed by researchers as a relatively stable trait that influences cognitive and behavioral tendencies and predicts behavior [8]. One of the most salient theories in this area is the Time Perspective Theory developed by Zimbardo and Boyd (2008) [9]. They argue that a person’s time perception influences decision-making, with the primary psychological impact located within present, past, or future timeframes [10]. As per this concept, Zimbardo and Boyd (1999) [11] constructed the Zimbardo Time Perspective Inventory (ZTPI), categorizing time perspectives into five distinct dimensions: Negative Past, Present Hedonistic, Future, Positive Past, and Present Fatalistic. Despite the rich theoretical framework, direct causal evidence connecting time perspectives to altruism or generosity remains scant. Nonetheless, some findings align with this possibility.For instance, humans are considered to contemplate the future more than any other species on Earth and share more resources with non-kin [12]. While most, if not all, other animals appear to lead lives centered around the present [13], and experience sampling studies have documented humans’ frequent “mental time travel” to the past and future [14]. Moreover, a substantial portion of human psychological processes seems to be prospective and pragmatic, focusing on actions necessary in the present to achieve positive future outcomes [15]. In recent work, Sjåstad (2019) [16] found that a decision-maker’s time perspective is critical in social decision-making, with future-oriented thinking potentially fostering reputation-based generosity. This suggests that one of the primary functions of human foresight might be to stimulate voluntary resource sharing, thereby promoting cooperation within groups and societies.

Research confirms that time perception is one of the key factors affecting intertemporal decision-making [17]. To highlight variations in individual perceptions of time duration, Zauberman et al. (2009) [18] introduced the parameter “time sensitivity”, proposing the Perceived–Time–Based Model (PTBM). This model posits that perceiving a time duration as shorter can reduce the waiting time for delayed rewards, thereby demonstrating greater patience and a preference for delayed benefits. Conversely, viewing a time duration as longer can increase individuals’ impulsivity and amplify the risks of “waiting” [19,20], leading people to choose immediate benefits. Simultaneously, the perception of time duration can also impact how individuals evaluate the magnitude of benefits. For instance, a longer estimate could potentially lessen the perceived value of future benefits [21]. Furthermore, individual differences in time perception affect delay discounting. For shorter delay times, varying levels of time sensitivity lead to non-linear changes in discount rates in group research data [22]. These insights indicate that individuals’ perceptions of time may influence intertemporal prosocial behavior. Considering that people’s time perspective is a relatively stable personality trait and their temporal orientations toward the past, present, and future are emphasized differently [23], it may then impact intertemporal prosocial decisions. In practical terms, some people make decisions expecting future reciprocation, trading early benefits for delayed costs. Others, however, prioritize immediate altruistic satisfaction and concern themselves with immediate benefits. This leads to an intriguing question: What preference characteristics do individuals with differing time perspectives display when confronted with intertemporal prosocial choices?

Regrettably, research directly investigating the relationship between time perspective and intertemporal social discounting is scarce, leading to a lack of comprehensive theoretical backing and detailed study [16]. Consequently, our study delves into the intertemporal prosocial choice characteristics of individuals with divergent time perspectives, aiming to decipher how time perspective impacts the mechanisms of intertemporal prosocial choices. Moreover, our research also probes whether the influence of time perspective traits on intertemporal prosocial discounting occurs at the evaluation or comparison stage. For this purpose, we employed the recently developed Dictator Game with Delayed Rewards [24].The Chinese version of the Zimbardo Time Perspective Inventory [25] was utilized to categorize groups based on different time orientations, enabling an examination of the characteristics and disparities of participants’ behavior preferences in intertemporal prosocial choices.

Drawing upon to the PTBM [25,26,27], our hypotheses are as follows:

**Hypothesis** **1.** *Individuals with a Present Impulsive Time Perspective (PITP) are likely to favor immediate altruistic satisfaction in intertemporal prosocial choices.*

**Hypothesis** **2.** *Individuals with a Future Time Perspective (FTP) might prefer delayed altruistic satisfaction.*

Therefore, Thus, we propose that when participants are temporally distant from the beneficiary population, individuals with a PITP would significantly decrease generosity, which is less pronounced among FTP individuals.

## 2. Materials and Methods

### 2.1. Participants and Measurement

The study utilized the validated ZTPI-C [25]. The ZTPI-C consists of 25 items, and its factor structure is similar to the original scale. The authors of ZTPI-C pointed out that after revising the list, the “Present Hedonism” dimension only reflects impulsivity. Therefore, we also used “Present Impulsive” instead of “Present Hedonism”, which is related to the characteristics of impulsivity, carelessness, and disregard for consequences [25]. We selected participants with the three temporal traits of “Positive Past”, “Present Impulsive”, and “Future” from students (items are listed in the Supplementary Materials Table S1). Participants were asked to rate each item in terms of their characteristics on a 5-point Likert scale, ranging from 1 (“strongly disagree”) to 5 (“strongly agree”). To evaluate to what extent the scale items measure the same construct with freedom from random errors (internal consistency), we used a scale reliability measurement [28]. Following one of the Reviewer’s suggestions, the structural validity of ZTPI-C was evaluated by interpreting the Mokken scale based on non-parametric Item Response Theory [29]. The Mokken package [30] in the R programming environment was used for this analysis [31]. Referring to previously published approaches [32,33], we input the data obtained from the three subscales of ZTPI-C into the Mokken Scaling Program. The results showed that the three subscales, “Past Positive” (6 items), “Present Impulsive” (4 items), and “Future” (5 items), encompassing a total of 15 items, exhibited good scalability (item H coefficients 0.40–0.47) and formed a robust scale (total H coefficient 0.43). Item ordering was applicable for all participants. Furthermore, Cronbach’s alpha value for the “Future” subscale was 0.71, for the “Present Impulsive” subscale was 0.70, and for the “Positive Past” subscale was 0.76.

Since the ZTPI-C does not have the function of dividing types according to score dimensions, a data-driven K-clustering method was used [34]. The clustering results divided the participants into the Present-Impulsive Time Perspective (PITP) group; the Future Time Perspective (FTP) group (see Table 1). 82 right-handed individuals (43 males; average age 24.36 y, SD = 4.27) participated in this experiment. Due to its small size, the analysis did not include the Positive Past group (N = 5).

A priori sample size estimate was conducted using G*Power 3.1 software [35], with at least 56 participants required to achieve a standard power of 0.80 and an effect size of f=0.25. Therefore, the sample size of this study is adequate. This study was approved by the Ethics Committee of the Harbin Institute of Technology, and according to the Helsinki Declaration, all participants gave written informed consent before the experiment. Based on their decisions made during the intertemporal prosocial discounting task, they received a show-up fee of CNY (Chinese yuan) 30 (~$4) and a variable amount (see below).

### 2.2. Intertemporal Prosocial Discounting Task

**Figure 1:**
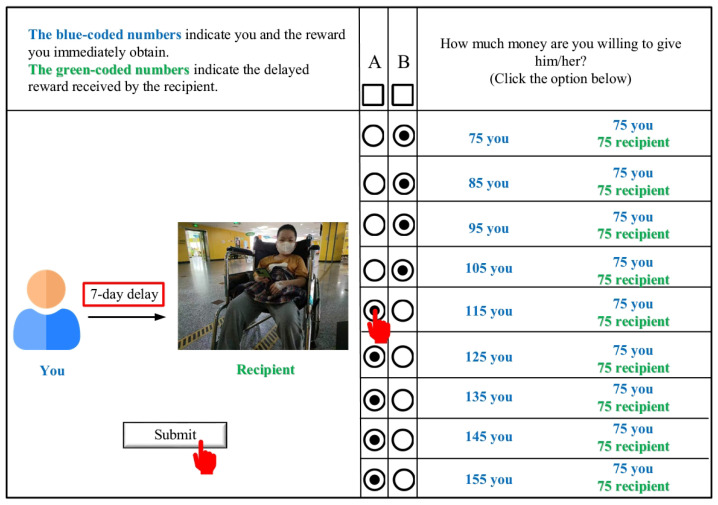
Illustration of a single trial during the intertemporal prosocial discounting task. Option A signifies a selfish choice, whereas Option B denotes a generous choice. The numbers coded in green represent the recipient and their reward magnitude, while the blue-coded numbers correspond to the participant.

The study utilized the Dictator Game with Delayed Rewards, a novel experiment recently proposed by Lu et al. (2022) [24]. The distinct aspect of this experiment lies in introducing a temporal delay between the decision-making time (during the experiment) and the payment time (our manipulation) by postponing the payment. Seven-time delay manipulations were implemented (in days), specifically 1, 5, 7, 14, 30, 60, and 100 days. In our modified task, participants were notified that they would observe the faces of 7 fundraisers on the screen and would need to decide how much money they were willing to donate to each. Participants were prompted to consider how their donation behavior would be altered when different anonymous fundraisers received the donations at varying time delays (see Figure 1).

In the main experiment, for each face presented, participants had to choose between (A) a selfish option or (B) a generous option: (A) the selfish option entailed the participant receiving a variable, larger sum of money for themselves (with nine possibilities, ranging from CNY 75 to 155 in increments of CNY10 throughout the trial), while (B) the generous option meant that the participant and the recipient would receive a fixed sum of CNY 75 each. We adopted the same amounts as those Vekaria et al. (2017) [36] used in their social discounting task. The task comprised seven blocks, each representing a fundraiser at a specific delay. Within each block, the order of selfish amounts was randomized, and the sequence of the blocks was also randomized. The block design aims to reduce the cognitive load incurred by individuals when switching between different delay levels [37]. The experiment was programmed in JavaScript using jsPsych [38].

Notably, the delay only applies to the recipient, with participants receiving their portion of the money immediately. Secondly, to avoid participants alternating between generous and selfish options multiple times, the experimental webpage was designed to consolidate their decision with a single click. For example, if a participant clicks on Option A between [A] CNY 115 and [B] CNY 75/75, the Option A checkbox is automatically filled for all options where [A] > CNY 115, and for all options where [A] < CNY 115, the Option B checkbox is filled. If participants wished to choose only selfish or generous options at a specific time delay, they could check either A or B in the “Select All” box, respectively. After selecting, participants press the “Submit” button to go to the next question. The details of the experiment are provided in the Supplementary Materials.

### 2.3. Procedure

All participants were welcomed with a standard greeting, inviting them to participate in the research and informing them that their contributions would support an online charity. To begin the experiment, we presented a real charity donation scenario to the participants, designed to familiarize them with the concept of time delay used in the task (see the Supplementary Materials for details). Participants were encouraged to immerse themselves in the decision-making process outlined in the scenario, making choices based on their actual circumstances. Participants were further informed that each fundraiser would receive their designated donation after a specific delay (note that this was not deception; we did exactly that) to ensure they made each choice seriously. Participants were given a participation fee of CNY 30. At the end of each experiment, one trial was randomly selected, and participants would receive an additional payment equal to 30% of the value of the actual decision. Thus, the experiment was conducted in an incentive-compatible manner, involved no deception, and adhered to the standards of economic research [39].

### 2.4. Discounting Data Analysis

We determined the indifference points for each delay level between selfish and generous options. Details regarding the calculation of indifference points are provided in the Supplementary Materials. The net amount forgone was determined by subtracting CNY 75 (the rewards participants would earn with the generous option) from the indifference points, representing the actual cost of relinquishing resources. We then utilized the amounts participants were willing to forgo as a measure of generosity. According to Construal Level Theory [40], temporal distance and social distance are both dimensions of psychological distance, and both have similar effects on decision making.Therefore, we followed previously established data analysis methods [37]. Two primary approaches exist for analyzing discounting data: calculating the discounting slope by fitting a discount function informed by theory or calculating the area under the curve (AUC) [41]. The AUC was calculated for each participant by normalizing each delay and the amount forgone, drawing vertical lines from each data point to the x-axis, and summing the series of trapezoids formed. Consequently, AUC values range from 1 (representing no discounting) to 0 (representing maximal discounting). An independent sample *t*-test was used to compare AUC values between the PITP and FTP groups.

Subsequently, we evaluated generosity as a function of time delay using an intertemporal prosocial discounting model [24]. We fitted the amount forgone at each delay to the standard hyperbolic model, $v=\frac{V}{1+kT}$, where *V* denotes the undiscounted value of the reward, *k* signifies the degree of discounting, *T* represents time delay, and corresponds to the actual discounted value of the reward. In addition, the free parameters *V* and *k* imply the subjective value of generosity towards short-term donation targets and the steepness of decline in generosity across delay levels separately. The fitted exponential parameters for both groups were also calculated, and the analysis revealed a lower model fit (refer to Supplementary Materials Table S2).

## 3. Results

### 3.1. Time Delay Reduces Generosity

**Figure 2:**
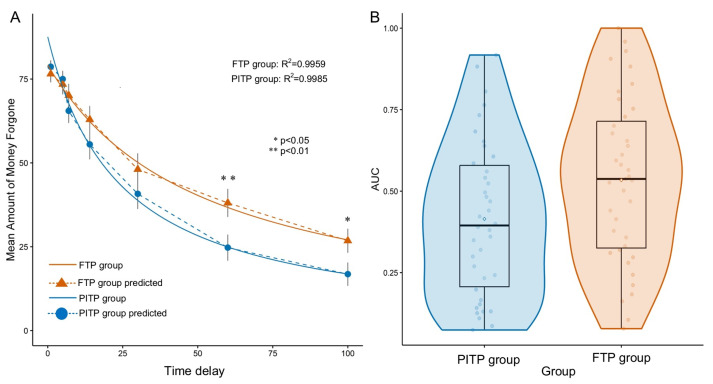
Intertemporal prosocial discounting and AUC for the PITP and FTP groups. (**A**). The intertemporal prosocial discounting curves (mean of all participants, N=77) are plotted as the maximum amount willing to forgo (*v*) versus time delays (*T*) from 1 to 100. The prediction curves illustrate the better-fit hyperbolic functions for the PITP and FTP groups. Asterisks denote delays (T = 60, 100 days) at which a significant difference was observed between the two groups in the amount they were willing to forgo, as per the independent sample *t*-test. Error bars represent the standard error of the mean (s.e.m.). * p<0.05, ** p<0.01. (**B**). AUC box-whisker contour plots for the PITP and FTP groups are shown. Light-colored dots represent the group means. The whiskers display the data range (minimum and maximum values). The band within the box represents the second quartile (median of AUC). The boxes represent the third (**top**) and first (**bottom**) quartiles, and the shaded areas depict the relative percentage (frequency) of participants with corresponding AUC values.

As anticipated, the amount of money participants was willing to forgo significantly decreased as time delay increased, b=−0.56, SE=0.02, t=−25.52, p<0.001, $R^{2}=0.67$. This effect was evident in both the PITP group (b=−0.61, SE=0.03, t=−17.86, p<0.001, $R^{2}=0.64$) and the FTP group (b=−0.51, SE=0.03, t=−18.64, p<0.001, $R^{2}=0.71$) (see Figure 2A). This suggests that generosity decrease as the delay increases, regardless of time perspective.

### 3.2. Different Time Perspectives and Generosity

Initially, we compared the two free parameters, *V* and *k*, across the two groups. A Mann-Whitney-Wilcoxon test revealed a significant difference between the PITP group (Median=0.031) and the FTP group (Median=0.021) in terms of *k*, z=2.30, p=0.021, effect size r=0.26. The *V* parameter, representing the height of the discount function, did not find a significant difference between the two groups (z=1.06, p=0.289).

Next, results of an independent sample *t*-test indicated that greater AUC for the FTP group (M=0.53, SD=0.25) than the PITP group (M=0.41, SD=0.24; t(75)=2.14, p=0.036, Cohen’s d=0.49, 95% CI [0.03,0.94]). The robust effect size is Cliff’s delta=−0.27, 95% CI [−0.49,−0.02]. Together, the results yield a small effect size. These also confirmed different intertemporal prosocial discounting between the FTP and PITP individuals (see Figure 2B).

Finally, we conducted a 2 (type of time perspective: PITP vs. FTP) × 7 (delay levels) mixed ANOVA, with the type of time perspective as a between-subjects factor, delay level as a within-subject factor, and the average amount of money forgone as the dependent variable. The results showed a significant interaction between time perspective and time delay, F(3.22,241.82)=2.91, p=0.032, $\eta_{p}^{2}=0.037$ (Greenhouse-Geisser corrected). In a simple effects analysis, individuals with a PITP exhibited significantly smaller amounts of money forgone at large time delays (60 and 100), t(75)=2.70, p=0.009, Cohen’s d=0.68, 95% CI [0.18,1.12]; t(75)=2.35, p=0.021, Cohen’s d=0.53, 95% CI [0.08,0.99]. We also calculated a robust effect size for large time delays (60 and 100), Cliff’s delta=−0.36, 95% CI [−0.57,−0.10]; Cliff’s delta=−0.34, 95% CI [−0.55,−0.09]. Following previous studies [42,43], these results indicated a medium effect. When comparing the level of generosity at each delay level, we discovered a significant influence of temporal insight on several delays. There was a significant difference between the FTP and PITP groups between 60 and 100 days of delay, as shown in Figure 2A. Our results suggest that individuals in the FTP group demonstrated greater generosity towards recipients with larger delays, while the level of generosity in individuals from the PITP group was much lower. Therefore, FTP individuals are more inclined towards delayed altruistic satisfaction than those with a PITP.

### 3.3. Time Perspective Predicts Intertemporal Prosocial Preferences

**Figure 3:**
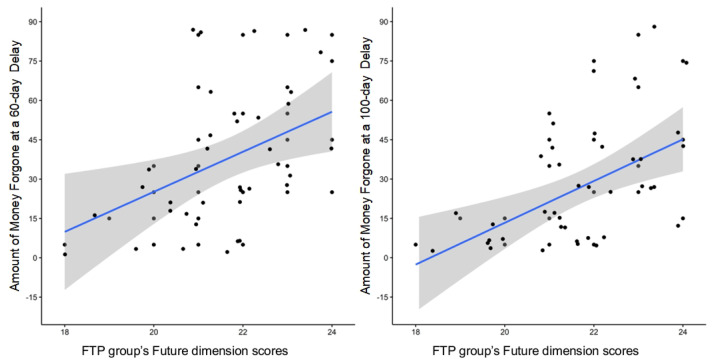
The FTP individuals’ scores on the “Future” dimension positively correlate with the amount of money forgone. The shaded regions indicate the 95% confidence interval.

To examine the predictive effect of time perspective on intertemporal prosocial discounting, we calculated Spearman’s correlations between the amounts of money forgone by participants in each group and their ZTPI-C scores. Analyses revealed that the correlations for participants in the PITP group were insignificant (all p>0.05). A similar procedure was performed for the FTP group, yielding positive correlations on the “Future” scores (delay = 60 days, RHO=0.41, p=0.010; delay = 100 days, RHO=0.53, p=0.001, see Figure 3), but non-significant correlations on the “Present Impulsive” scores (all p>0.05). The results of the individual differences analysis in the FTP group demonstrated a positive correlation between the amount of money forgone and the scores in the “Future” dimension. This indicates that for individuals with an FTP, “Future” scores of the ZTPI-C can predict intertemporal prosocial preferences, particularly under larger time delays.

## 4. Discussion

In this study, we adopted a novel delayed reward dictator game and applied the ZTPI-C to categorize two groups: one with a PITP and the other with an FTP. We explored their preference characteristics and mechanisms in intertemporal prosocial decision-making. Consistent with prior research findings [24,44], we discovered that both groups were willing to forgo a certain amount of money for others’ benefit, and their degree of generosity decreased as the time delay increased. The discounting behaviors of both groups could be effectively explained by a standard hyperbolic function.

Further analysis indicated that time perspective influenced how generosity changed with the increase in delay. Initially, we identified the main effect of time perspective on generosity across various delay levels. Second, individuals with an FTP exhibited a lower discount rate and a stronger preference for delayed altruistic satisfaction than those with PITP. Regarding the main effect, significant differences in generosity between the two groups emerged when the time delay reached 60 and 100 days. Finally, the FTP group demonstrated a positive correlation between their scores on the “Future” dimension of the ZTPI-C and the amount of money forgone. As a result, overall, FTP individuals demonstrated less variability than PITP individuals in other-regarding generosity, maintaining significantly closer to the most financially efficient outcomes. This demonstrated that, in the context of intertemporal charitable donations, FTP individuals were more inclined to adopt a rational and careful attitude towards selfish temptations than those with a PITP. They expected to receive reciprocation from others in the future and were willing to exchange early benefits for delayed costs. This finding was backed by previous research: FTP individuals demonstrated stronger achievement motivation closely associated with goals [45]. Zaleski and Przepiórka (2015) [46] found that FTP individuals had higher self-efficacy and more remarkable persistence. Additionally, Taylor and Wilson (2016) [47] reported that FTP individuals invested more time and effort into achieving long-term goals and maintained strong beliefs. Our findings extended the above conclusions to the prosocial domain, suggesting that, in the long run, individuals with a high degree of future orientation were more inclined to choose options that could bring benefits or advantages to others. Therefore, as a relatively stable personality trait, time perspective could distinctly differentiate individuals’ intertemporal prosocial behavioral preferences, thereby predicting selfish or generous behavioral outcomes.

Research on human intertemporal choices confirmed that the evaluation and comparison stages are crucial phases that promote decision-making and reflect its core processes, which have a causal impact on choices [48]. Accordingly, we addressed the second question: At what stage does time perspective influence intertemporal prosocial choices—evaluation or comparison? Our study showed that compared to individuals with a PITP, those with an FTP tend to favor delayed altruistic satisfaction. This phenomenon can be entirely explained from the perspective of the PTBM. People often overestimate the abundance of future resources, especially time. When an event is imminent, time becomes scarcer than money and thus has greater value, resulting in a decline in value with the delay of benefits [49]. Additionally, research has confirmed that increases in time compression levels and decreases in time sensitivity can cause changes in the magnitude of hyperbolic discounting [19]. We inferred that the hyperbolic discounting mechanism in intertemporal prosocial choices might be due to a decrease in sensitivity to delay levels: as the delay increases, individuals’ sensitivity to delay levels gradually decreases; an overall increase in time compression levels also affects the level of hyperbolic discounting. Individuals with different time perspectives assign different costs to present and future time, so their sensitivity to perceived time levels also differs. Following this line of thought, individuals with an FTP focus on the future, taking the “Future” as the reference point, and the perception of delay time is shortened. As a result, hyperbolic discounting declines more slowly and tends towards delayed altruistic satisfaction. However, individuals with a PITP focus on the present, taking the “Present” as the reference point, and subjectively the delay time is longer. Therefore, their hyperbolic discounting declines rapidly, indicating a preference for immediate altruistic satisfaction. Thus, we have reason to believe that the impact of time perspective on intertemporal prosocial choices occurs in the evaluation stage.

There are several limitations in the current research. First, generosity is a cultural construct bound by cultural constraints [50]. However, our research was conducted within the scope of Chinese culture and values. As such, the findings on time insight and delay affecting charitable donations may vary in different cultures. Future research should consider cultural differences in the context of intertemporal altruism. Second, the convenience sample drawn for this study was highly educated. All participants were students, which may limit the generalizability of the results to other populations. This is particularly noteworthy considering that older adults often exhibit more positive reactions to helping others [51], a factor we could not control. Therefore, future research requires larger samples and more rigorous procedures to validate these findings further. Lastly, we only studied one form of prosocial behavior. The advantage of the dictator game with delayed rewards is that intertemporal altruistic behavior can be objectively and accurately measured. Future research should confirm whether our findings can be extended to other forms of prosocial behavior.

Drawing on the theory of the PTBM, this study compared the preference characteristics of individuals with varying time perspectives in intertemporal prosocial decisions. The results illuminate how time perspective on intertemporal altruism: cognitive and attitudinal orientation towards time shapes an individual’s subjective time perception and evaluation. Moreover, the time perspective influences intertemporal prosocial behavior by modifying the discount level during the evaluation stage. This study contributes a fresh perspective toward understanding individual variances in intertemporal altruism and supplies novel empirical evidence for the inherent disparities in intertemporal prosocial decision-making. Additionally, it holds practical value in aiding individuals to comprehend their time perception and improve intertemporal charitable donations.

## 5. Conclusions and Policy Implications

The study suggests that, compared with FTP individuals, PITP individuals have a higher intertemporal prosocial discount rate and prefer immediate altruistic satisfaction. FTP individuals’ scores on the “Future” dimension of the ZTPI-C correlate positively with the amount of money they are willing to forgo; individuals with a stronger future orientation tend to be more generous. Therefore, time perspective, as a personality trait, can predict individual intertemporal prosocial preferences.

Moreover, traits can serve both as relatively stable predictors of success and viable targets for policy change and intervention [52]. Hence, for PITP individuals, policies could attempt to provide immediate feedback and rewards to stimulate their prosocial behavior. For instance, the government could establish public welfare lottery activities, where donations of a certain amount allow participation in the lottery, thereby attracting these individuals to engage. For FTP individuals, policies could emphasize prosocial behaviors’ long-term impact and value. For example, the government could develop education policies that educate the public on how prosocial behavior helps the long-term development of society, thereby encouraging them to engage in more prosocial behavior.

Overall, understanding the type of an individual’s time insight and formulating policies can more effectively motivate people to engage in prosocial behavior. This requires policymakers to deeply understand individuals’ psychological traits to formulate more effective and targeted policies.

## Figures and Tables

**Table 1:** The composition of participants and mean scores of ZTPI-C’s Present Impulsive and Future subscales.

	Group 1 (N = 38)	Group 2 (N = 39)
Present Impulsive	4.17	2.87
Future	2.58	4.43

## Data Availability

The data shown in this research are available on request from the corresponding author.

## Supplementary Materials

The following supporting information can be downloaded at: https://www.mdpi.com/article/10.3390/bs13070590/s1, Figure S1. Example of an intertemporal prosocial discounting task; Figure S2. Example of the intertemporal prosocial discounting task with a complete selection of generous and selfish options; Table S1. Items of the Chinese Version of the Zimbardo Time Perspective Inventory; Table S2. Discounting parameters for hyperbolic and exponential models. References [24,38,39,53,54] are cited in the supplementary materials.

## Abbreviations

The following abbreviations are used in this manuscript:

PITP	Present Impulsive Time Perspective
FTP	Future Time Perspective
ZTPI-C	the Chinese version of the Zimbardo Time Perspective Inventory
PTBM	the Perceived-Time–Based Model
