# Supplementary material for: Time Matters: Time Perspectives Predict Intertemporal Prosocial Preferences
Source: Behav Sci (Basel). 2023 Jul 14;13(7):590. doi: 10.3390/bs13070590 (PMC10376203; doi:10.3390/bs13070590)
Supplement: Supplementary file 1 [file behavsci-13-00590-s001.zip › behavsci-2485290-supplementary.pdf]

behavioral sciences

MDPI

Time matters: Time perspectives predict intertemporal prosocial preferences

Electronic Supplementary Material

These ESMs are associated with the paper “Time matters: Time perspectives predict intertemporal prosocial preferences” published in *Behavioral Sciences*.

1. Experiment: Method

1.1. Familiarization with the concept of time delay

Please read the following scenario:

In May 2008, a devastating earthquake occurred in Wenchuan, Sichuan Province, China, resulting in the highest number of casualties since the Tangshan earthquake. This catastrophe triggered a robust public response, with donations from China and worldwide accumulating to over CNY 50 billion. These donations contributed to the reconstruction efforts. Many Chinese volunteers and international humanitarian rescue teams participated in the disaster relief efforts. Throughout the post-earthquake recovery and reconstruction process, the donations supported education, housing, ecological preservation, and the relocation of affected communities. However, a time lag emerged between accepting and distributing donations due to the time needed to raise funds, allocate recipients, and determine donation usage. Donors might alter their donation behavior if delays in delivering donations to disaster victims become excessive. Likewise, fundraising projects targeting long-term reconstruction in earthquake-affected areas encounter a similar challenge. While these fundraising initiatives enhance long-term welfare, they may impact short-term welfare. Consequently, individuals prioritizing timely aid might decrease their donations to these projects.

In this study, we conducted an intertemporal analysis of prosocial donations to explore the changes in Chinese adults’ donation behavior when charitable recipients (anonymous fundraisers and charitable organizations) experience time delays in receiving donations. We operationalized time delays between participants and fundraisers by postponing payments in the dictator game. The subsequent section introduces the intertemporal prosocial discounting task.

1.2. Intertemporal prosocial discounting task

During the experimental sessions, participants undertook a task involving donations to an online charity platform. To measure intertemporal prosocial discounting, we chose a modified version of the dictator game, as recently implemented by Lu et al. (2022). In our adapted version of the task, participants were informed that they would see seven faces on the screen, each representing a fundraiser. The novelty of this experiment was to postpone the payment, thus creating a time delay (in days) between decision-making (experiment) and the payment (our manipulation variable). The seven target fundraisers were associated with the following time delays: 1, 5, 7, 14, 30, 60, and 100 days. Participants were asked to contemplate how their donation behavior would change when various anonymous recipients received donations within their corresponding time delays (1 day, 5 days, 7 days, 14 days, 30 days, 60 days, and 100 days) (see Figure S1).

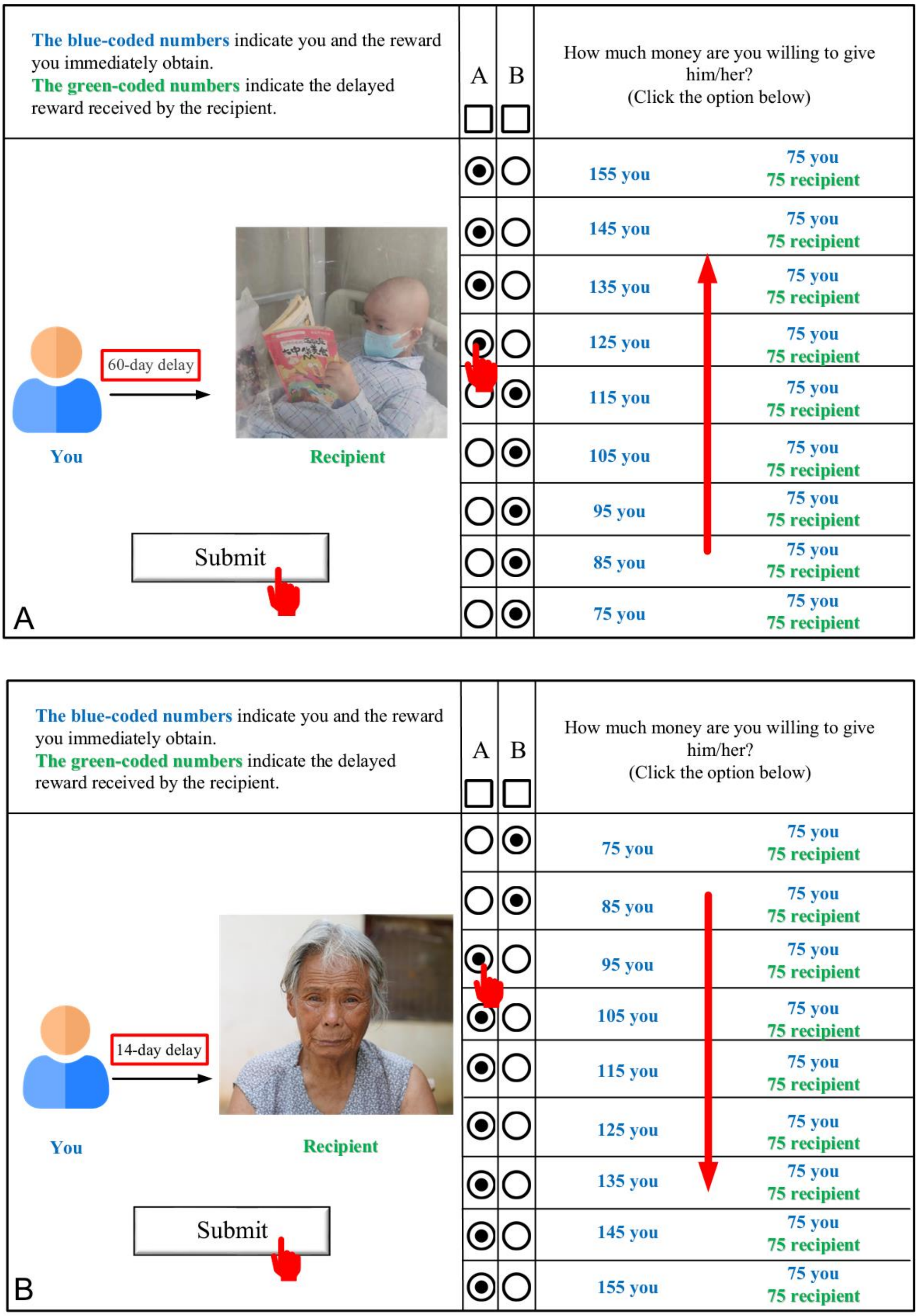
The blue-coded numbers indicate you and the reward you immediately obtain.
The green-coded numbers indicate the delayed reward received by the recipient.
A B
How much money are you willing to give him/her? (Click the option below)
60-day delay
You
Recipient
Submit
A
155 you
145 you
135 you
125 you
115 you
105 you
95 you
85 you
75 you
75 you
75 recipient
The blue-coded numbers indicate you and the reward you immediately obtain.
The green-coded numbers indicate the delayed reward received by the recipient.
A B
How much money are you willing to give him/her? (Click the option below)
14-day delay
You
Recipient
Submit
B
75 you
85 you
95 you
105 you
115 you
125 you
135 you
145 you
155 you
75 you
75 recipient

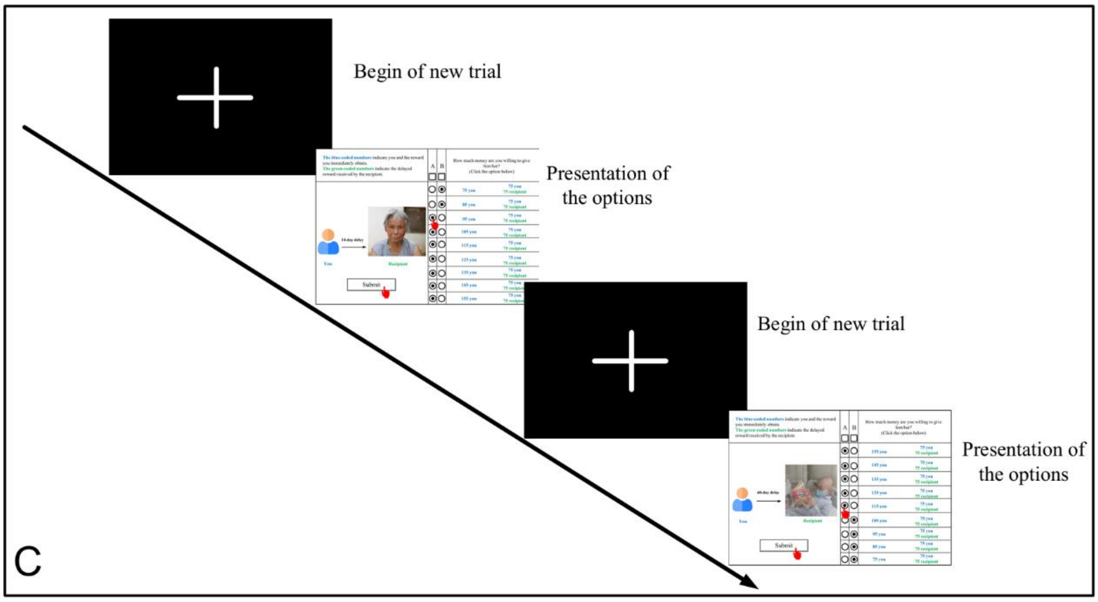

Figure S1. Example of an intertemporal prosocial discounting task. In each trial, two screens are presented. The first screen signals the commencement of the trial. The second screen presents two options: Option A symbolizes a selfish choice, and Option B symbolizes a generous choice. Green-coded numbers represent the recipient and the reward amount, while blue-coded numbers represent the participant. Participants were instructed to make nine choices on each page, arranged in columns. In the left column, exclusively, the participant receives money, ranging from CNY 75 to 155, in increments or decrements of CNY 10 (the order, either ascending or descending, is maintained for each participant). The right column specifies that both the participant and the anonymous recipient will receive CNY 75 each.

We informed the participants that they would see seven faces on the screen, one after another, in a balanced order. For each face, participants had to choose between selfish and generous options, either giving themselves a larger reward or giving themselves and the recipient a smaller reward. The selfish option varied in increments of CNY 10 across different trials, ranging from CNY 75 to 155. The generous option had a fixed CNY 75 for the participant and the recipient (see Figure S1). Note that delayed rewards were applied only to the recipient. Participants were aware that they would receive the remaining money immediately. This is quite natural, as donations typically come from the donor’s income, and recipients require time to accept the donations. Crucially, participants were additionally informed that the experimental webpage enabled them to indicate their preferences with a single click. The program determined a unique indifference point by preventing multiple preference switches. We use the trial in Figure S1(B) as an example. In this trial, the time delay was 14 days. If the participant clicked A between Options [A]95 and [B] (75/75), checkboxes for Option A would be automatically filled for all rewards >95. Simultaneously, for all rewards <95, Option B would be automatically filled. If participants desired to choose only selfish or generous options within a specific delay, they could check A or B, respectively, in the “Select All” box (see Figure S2). After making their choices, participants clicked the “Submit” button to advance to the next question. This task was written in JavaScript using the jsPsych toolbox (de Leeuw, 2015 [38]).

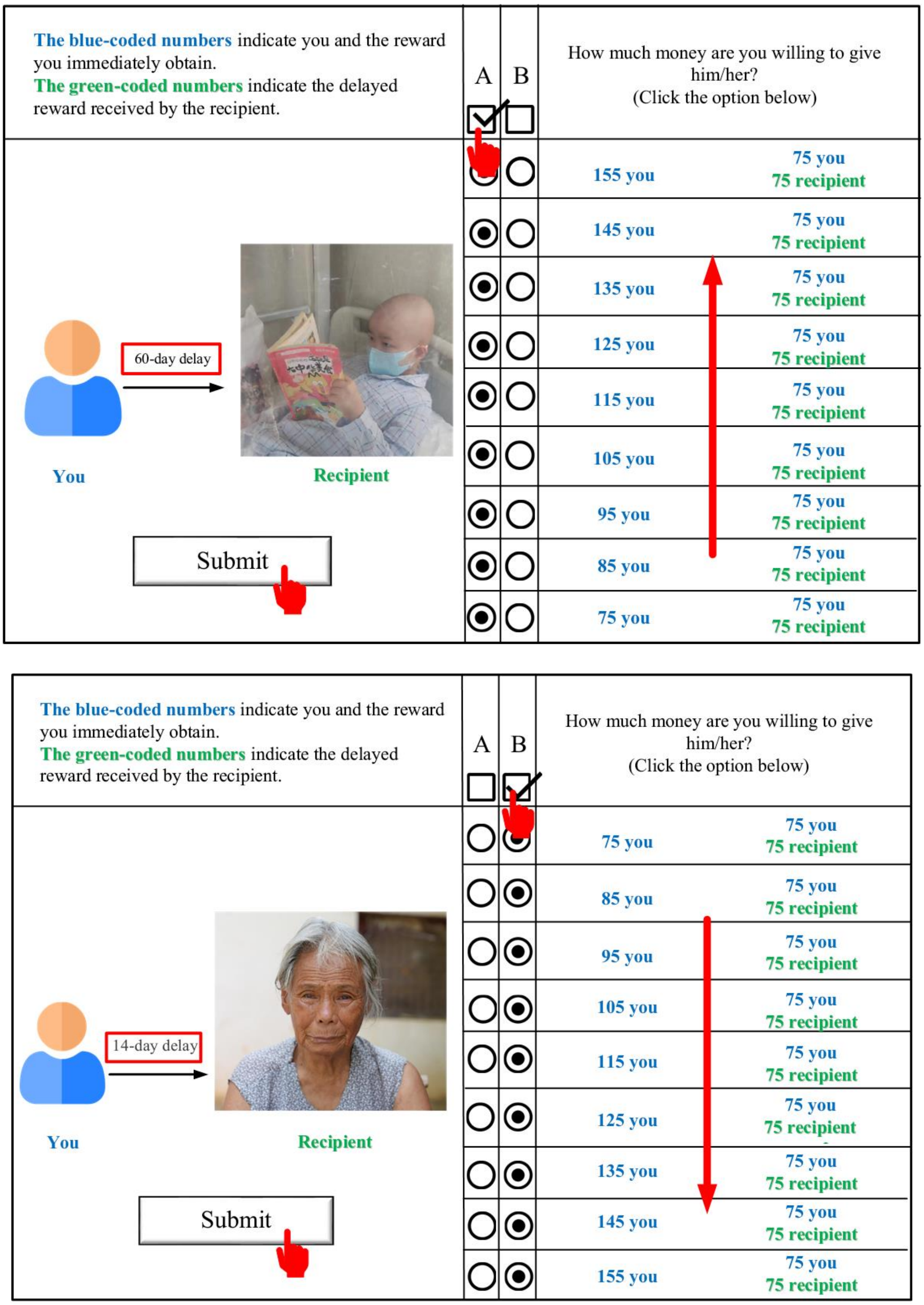

Figure S2. Example of the intertemporal prosocial discounting task with a complete selection of generous and selfish options.

The faces presented in the experiment were evenly distributed among young (7–29 years), middle-aged (33–59 years), and elderly (60+ years) individuals, and further divided by gender to better represent the diverse composition of the data collected. Referring to the Chicago Face Database (Ma, et al., 2015 [53]), these faces were presented with neutral expressions. To better engage participants, the experiment followed Christov-Moore and Iacoboni (2016) [54]. We told participants that all the faces represented real fundraisers,

and each fundraiser would receive the amount designated by the participant after a specific delay (note that this was indeed the case, so no deception was involved). Moreover, to increase the salience and relevance of participants' choices and align our experiment with incentives, participants received a CNY 30 participation fee. At the end of each experiment, a single trial was randomly selected from the experiment, and participants received an additional payment equivalent to 30% of the actual decision value. That is, if the participant chose the selfish option under the 14-day delay, they would receive a reward between CNY 22.5 and 46.5, depending on the selected trial. If the participant decided to be generous, they would receive CNY 22.5, and the other person would receive CNY 22.5. This payment rule was communicated to the participants before the start of the formal experiment. Therefore, our study did not involve deception and adhered to the standards for economic research (Schram, 2005 [39]).

1.3. The Chinese version of the Zimbardo Time Perspective Inventory (ZTPI-C)

Table S1. Items of the Chinese Version of the Zimbardo Time Perspective Inventory.

Copyright: © 2023 by the authors. Licensee MDPI, Basel, Switzerland. This article is an open access article distributed under the terms and conditions of the Creative Commons Attribution (CC BY) license (https://creativecommons.org/licenses/by/4.0/).

**Chinese**	**English**
1.熟悉的童年画面、声音和气味常常能勾起我美好的回忆	Familiar childhood sights, sounds, smells often bring back a flood of wonderful memories
2.回忆过去让我感到快乐	It gives me pleasure to think about my past
3.我做事冲动	I do things impulsively
4.我想完成某件事时，会设立目标并考虑达到目标的具体途径	When I want to achieve something, I set goals and consider specific means for reaching those goals
5.总的来说，我对过去生活的回忆中好事多于坏事	On balance, there is much more good to recall than bad in my past
6.在今晚玩耍之前，我会做完明天要截止的事情以及其他必要的工作	Meeting tomorrow's deadlines and doing other necessary work comes before tonight's play
7.我喜欢那些发生在过去的美好往事	I enjoy stories about how things used to be in the "good old times"
8.过去的痛苦经历在我的脑海中反复出现	Painful past experiences keep being replayed in my mind
9.有关美好时光的记忆常常浮现在我的脑海里	Happy memories of good times spring readily to mind
10.我总能按时兑现对朋友和上级的承诺	I meet my obligations to friends and authorities on time
11. 我凭一时冲动做决定	I make decisions on the spur of the moment
12.过去有太多不愉快的记忆，我不愿去想	The past has too many unpleasant memories that I prefer not to think about
13.我真希望过去的那些错误没有犯过	I've made mistakes in the past that I wish I could undo
14.我怀念童年的时光	I get nostalgic about my childhood
15.大多数事情都不能如我所愿	Things rarely work out as I expected
16.我很难忘记年少时那些令人不快的景象	It's hard for me to forget unpleasant images of my youth
17.由于时事变化无常，因此无法规划未来	You can't really plan for the future because things change so much
18.我的生活道路受制于某些我无法控制的力量	My life path is controlled by forces I cannot influence
19.我认为担忧未来毫无意义，因为你根本无能无力	It doesn't make sense to worry about the future, since there is nothing that I can do about it anyway
20.我通常能够循序渐进地按时完成计划	I complete projects on time by making steady progress
21.我常常跟着感觉走，而不是理性思考	I often follow my heart more than my head
22.我发现自己在兴奋时会忘乎所以	I find myself getting swept up in the excitement of the moment
23.我常想起那些曾经发生在我身上的坏事情	I think about the bad things that have happened to me in the past
24.只要有助于自己进步，我能坚持完成困难、枯燥的任务	I keep working at difficult, uninteresting tasks if they will help me get ahead
25.我常常想那些生活中错过的好事情	I think about the good things that I have missed out on in my life

Note. Each item should be rated on a 5-point scale: 1 = extremely uncharacteristic of me, 2 = somewhat uncharacteristic of me, 3= neither uncharacteristic nor characteristic of me, 4 = somewhat characteristic of me, 5= extremely characteristic of me. Past negative: 8, 12, 13, 15, 16, 23, 25; past positive: 1, 2, 5, 7, 9, 14; present impulsive: 3, 11, 21, 22; present fatalistic: 17, 18, 19; future: 4, 6, 10, 20, 24.

2. Supplementary data results

2.1. Check for order effects

As the discounting pages were presented in a random order, we examined and addressed the possibility of order effects. For a given sequence of pages (e.g., 4637152), the differences between page numbers (+2, -3, +4, -6, +4, and -3) were calculated and summed to obtain the order summary score (-2). The results indicated that the order summary scores were independent of the possible variables of interest (time perspective, AUC, gender, age, and EQ60) through correlation analyses and t-tests (all $p > 0.17$). Due to these reasons, order effects were not further considered in the subsequent analyses.

2.2. The estimation results from the discounting models

We calculated the subjective indifference point by titrating the magnitude of the selfish reward. The indifference point represented the point where the selfish and generous options had equal subjective value. The actual cost of choosing generously was calculated by subtracting CNY 75 (the amount participants received with the generous option) from the indifference point. With this, we identified the amount of money participants were willing to sacrifice at a particular delay. We averaged these values of the amount forgone across participants in each group for each delay level. We then adopted the hyperbolic discounting equation $v = \frac{V}{1+kT}$ and exponential discounting equation $v = Ve^{-kT}$ to fit the mean amount of money forgone separately for the present impulsive time perspective (PITP) and the future time perspective (FTP) groups. This yielded a hyperbolic model fit for both groups ($R^2_{\text{PITP}} = 0.9985$; $R^2_{\text{FTP}} = 0.9959$). Comparisons using Akaike's information criterion (AIC) and Bayesian information criterion (BIC) indicated that the hyperbolic model provided a better fit than the exponential model (Table 1).

Table S2. Discounting parameters for hyperbolic and exponential models.

Model	Group	Fitted parameters	Model fit	AIC	BIC
Hyperbolic model	PITP	$k = 0.04186$<br>$V = 87.5850$	$R^2 = 0.9985$	−77.2138	−77.3761
	FTP	$k = 0.01927$<br>$V = 79.1919$	$R^2 = 0.9959$	−79.8255	−79.9878
Exponential model	PITP	$k = 0.01588$<br>$V = 73.3621$	$R^2 = 0.9691$	−6.9244	−7.0867
	FTP	$k = 0.01066$<br>$V = 74.1233$	$R^2 = 0.9783$	−15.0666	−15.2289

*PITP (present impulsive time perspective); FTP (future time perspective).

References

38. de Leeuw, J. R. (2015). jsPsych: A JavaScript library for creating behavioral experiments in a Web browser. *Behavior Research Methods, 47*, 1-12.

24. Lu, T., Liang, D., Hong, M., & Sun, J. (2022). Regret Now, Compensate It Later: The Benefits of Experienced Regret on Future Altruism. *Frontiers in Psychology, 13*.

39. Schram, A. (2005). Artificiality: The tension between internal and external validity in economic experiments. *Journal of Economic Methodology, 12*, 225-237.

53. Ma, D. S., Correll, J., & Wittenbrink, B. (2015). The Chicago face database: A free stimulus set of faces and norming data. *Behavior Research Methods, 47*, 1122-1135.

54. Christov-Moore, L., & Iacoboni, M. (2016). Self-other resonance, its control and prosocial inclinations: Brain-

behavior relationships. *Hum Brain Mapp*, *37*, 1544-1558.

Disclaimer/Publisher's Note: The statements, opinions and data contained in all publications are solely those of the individual author(s) and contributor(s) and not of MDPI and/or the editor(s). MDPI and/or the editor(s) disclaim responsibility for any injury to people or property resulting from any ideas, methods, instructions or products referred to in the content.
